# Evaluation of the Activity of Choline Acetyltransferase From Different Synaptosomal Fractions at the Distinct Stages of Spatial Learning in the Morris Water Maze

**DOI:** 10.3389/fnbeh.2021.755373

**Published:** 2021-10-13

**Authors:** Zinaida I. Storozheva, Elena I. Zakharova, Andrey T. Proshin

**Affiliations:** ^1^V. Serbsky National Medical Research Centre for Psychiatry and Narcology, Moscow, Russia; ^2^Research Institute of General Pathology and Pathophysiology, Moscow, Russia; ^3^P.K. Anokhin Institute of Normal Physiology, Moscow, Russia

**Keywords:** spatial memory, choline acetyltransferase, water maze, hippocampus, neocortex

## Abstract

Accumulated data have evidenced that brain cholinergic circuits play a crucial role in learning and memory; however, our knowledge about the participation of neocortical and hippocampal cholinergic systems in spatial learning needs to be refined. The aim of this study was to evaluate the association of the activity of membrane-bound and soluble choline acetyltransferase (ChAT) in the synaptosomal sub-fractions of the neocortex and hippocampus with performance of the spatial navigation task in the Morris water maze at different temporal stages of memory trace formation. To identify distinct stages of memory formation, rats were trained using a 5-day protocol with four trials per day. The mean escape latency for each trial was collected, and the entire dataset was subjected to principal component analysis. Based on the Morris water maze protocol, there were three relatively distinct stages of memory formation: days 1–2, day 3, and days 4–5. The remotely stored memory trace tested in repeated and reversal learning beginning on day 19 (14 days after the end of initial learning) was associated at the individual level mainly with performance during the second trial on day 21 (the third day or repeated or reversal learning). The ChAT activity data suggest the participation of cortical cholinergic projections mainly in the first stage of spatial learning (automatic sensory processing) and the involvement of hippocampal interneurons in the second stage (error-corrected learning). Cholinergic cortical interneurons participated mainly in the stage of asymptotic performance (days 4–5). It is advisable to evaluate other signalling pathways at the identified stages of memory formation.

## Introduction

Brain cholinergic circuits play a crucial role in learning and memory, so evaluating the involvement of acetylcholine (ACh) signalling pathways in different stages of memory trace formation is important for understanding cognitive processes and the aetiopathogenesis of age-related dementia, Alzheimer’s disease and post-stroke mental disorders, among other conditions. Many researchers have evidenced the participation of ACh in the mechanisms of spatial navigation (Deiana et al., 2011). However, the role of hippocampal and cortical ACh in the acquisition, consolidation, storage, retention and adaptive flexibility of new, recent and remote memory traces in spatial learning models is still poorly understood. Moreover, the results obtained in different studies are controversial (Parent and Baxter, 2004; Deiana et al., 2011; Drever et al., 2011). This is largely due to the fact that the forebrain cholinergic system acts as a neuromodulator (Gold, 2003) and can contribute differently in the course of ongoing multiple mnemonic processes that provide unified space representation and an adaptive behavioural pattern.

In particular, selective loss of septohippocampal and/or corticopetal cholinergic projections has been shown to cause attention deficit (McGaughy et al., 1996; Lehmann et al., 2003). In turn, attention deficit can have a variety of effects on the encoding of new memories, especially, in the early stages of learning. Accordingly, the sufficient level of the activity of cholinergic circuits is needed during the beginning stages of the long-term memory formation. At the same time, a decrease in the level of acetylcholine contributes to the successful consolidation of the memory trace (Hasselmo and McGaughy, 2004; Micheau and Marighetto, 2011). Also, some data evidenced the possibility of the involvement of the forebrain cholinergic circuits in the mechanisms that supply the flexibility of memory trace including the transfer of acquired experience and learning rule across behavioural problems (Conner et al., 2003; Sarter et al., 2003). However, all these statements and hypotheses need further verification and development.

Multiple neurochemical and behavioural strategies of investigation should be applied to evaluate this issue. Alongside the variety of ACh receptors, the heterogeneity of the origin, structure and functional properties of cholinergic fibres both in the cortex and in the hippocampus should be considered. The main cholinergic source in the cortex is provided by projections from the basal forebrain nuclei. The existance of the large population of cholinergic interneurons in the cortex and their participation in mechanisms of memory trace formation also has been shown (Dudai et al., 2020). In the hippocampus, the bulk of ACh is found in projections from the diagonal band of Broca and the medial septum, but there are also intrinsic hippocampal cholinergic neurons (Frotscher et al., 2000; Yi et al., 2015). Based on previous studies (Mukhin et al., 2002; Zakharova et al., 2010b), the synaptic activity of cholinergic interneurons and cholinergic projection fibres can be estimated by the activity of choline acetyltransferase (ChAT) from two distinct fractions of synaptosomes: light (obtained mainly from projection fibres) and heavy (obtained mainly from interneurons). Other authors have emphasised that light and heavy synaptosomes display markedly different functional proteomics and metabolic machinery; these differences suggest their functional specificity (Gorini et al., 1999). In addition, the activity of two forms of ChAT, soluble and membrane bound (sChAT and mChAT, respectively), can be measured in each type of synaptosome. Hence, it is possible to study selective (in particular, tonic and phasic) mechanisms of regulations of cholinergic neurotransmission (Pahud et al., 2003). Alongside the increase in the selectivity of neurochemical methods, the delineation of the temporal stages of memory trace formation in multi-trial navigation models is advisable. The deconstruction of learning processes using different training protocols has been applied (Haider and Tabassum, 2018), but the application of multivariate statistical techniques would also provide additional insights.

Navigation in the Morris water maze (MWM) is widely used in animal studies of spatial memory and to estimate the activity of nootropic and neuroprotective drugs, especially in the animal models of Alzheimer’s disease (Vorhees and Williams, 2006; Wahl et al., 2017). It should be noted that, despite the seeming simplicity of this behavioural paradigm, a variety of mnemonic processes underscore the task solution. Successful navigation in a novel environment requires collecting information about environmental cues and their relative position (space mapping) as well as integration of this allothetic information with self-motion (idiothetic) signals. Researchers have shown the specific involvement of different brain networks into processing allothetic and idiothetic signals (Berger-Sweeney et al., 1994; Burgess, 2008). At the same, in the studies of acquisition, consolidation, reactivation and retention of unified space representation, the formation and interaction of multiple memory traces, i.e., the dynamic coupling between the activities of relevant brain structures as well as different patterns of neurotransmitter activity have been revealed (Arleo and Rondi-Reig, 2007). Moreover, as pointed out by Andre et al. (2019), “the distinct constellation of brain structures may participate to the space representation differently depending on the navigation strategy employed, the availability of sensory information and the phase of memory utilisation” (see also Sirota and Buzsàki, 2005). For example, in many studies the hippocampus has been shown to be a key player in processing allocentric information and spatial mapping of the environment, but recent studies have also revealed the participation of this structure in the control of self-generated motion (Poulter et al., 2019). Successful investigation of the dynamic pattern of brain regions and neurotransmitter circuit activity during the formation of unified space representation requires the identification of relatively distinct temporal stages of learning processes in the MWM. As noted above, several approaches could be applied here, including quantitative analysis of the performance dynamics and the application of multivariate statistical methods.

One of the most commonly used protocols in MWM includes training for 4–6 days with four trials each day (Terry, 2009). The different measures of memory formation and their dynamics are usually estimated in the frame of this model. Analysis of existing data shows that the daily mean escape latency – the time required to reach the platform – plateaus on days 3–4 (Terry, 2009; Haider and Tabassum, 2018; Ali et al., 2020). The escape latency in the first trial of each session is regarded as a putative measure of long-term memory; it becomes relatively stable on days 4–5. Morris and Frey (1997) paid special attention to the second trial of the first session as a measure of specific “one-trial” memory. The escape latency in the second to fourth trials on day 2 and beyond reflects “mixed” long-term and working memory.

We previously found correlations between different measures of spatial memory during a 3-day MWM training protocol and the activity of ChAT from different synaptosomal fractions (Zakharova et al., 2010a). The positive associations between the activity of ChAT from cortical synaptosomes and task performance were observed mainly during the early stages of memory formation whereas the activity of ChAT from hippocampal synaptosomes displayed negative correlations with task performance in the early and positive ones in the late stages of learning. In this study, we aimed to verify previous findings in a larger cohort of rats, and to estimate the impact of the learning protocol (number of training days) on the association between ChAT activity and spatial memory. Of note, the performance at several periods of acquisition, consolidation, reactivation and reminding in the frame of this protocol could contribute differently to the stability and flexibility of adaptive navigation strategy that is to be stored for a long time. Among the variety of possible approaches, a principal component analysis (PCA) could be helpful to clarify the temporal pattern of learning in MWM.

The goal of the present study was to detect the distinct stages of spatial learning in the MWM in the frame of a 5-day training protocol with four trials each day using PCA and to reveal the associations of sChAT and mChAT activity from different sub-fractions of hippocampal and neocortical synaptosomes with navigation performance at each of identified stages. To estimate physiological relevance of PCA results, we assessed the associations between task performance at different temporal points of initial learning and the measures of remote memory trace (retention, repeated learning, or reversal learning) observed 2 weeks after the initial learning.

## Materials and Methods

### Experimental Animals

Outbred white adult male rats (200–250 g) were obtained from the “Light mountains” animal nursery (Russia) and then kept in the vivarium of the Institute of Normal Physiology in a temperature-controlled room (21–22°C) with free access to food and water and a 12-h photoperiod. All experimental protocols were performed according to the National Institutes of Health Animal Care and the “Principles of Laboratory Animal Care” guidelines, and the study was approved by the Ethical Committees of the relevant institutes.

### Behaviour Tests

**FIGURE 1:**
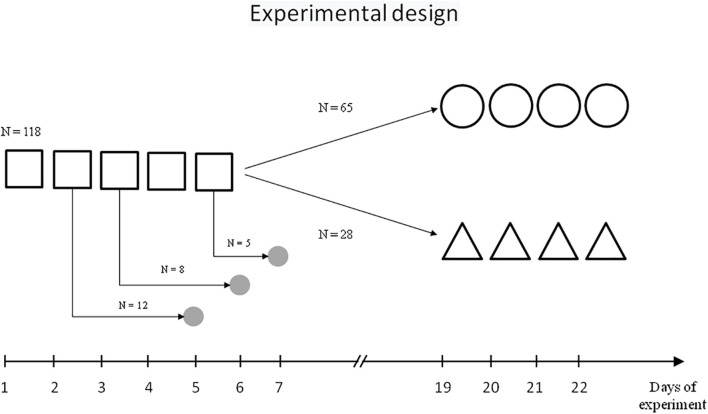
Scheme of experiment. 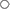
– initial learning, 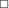
– repeated learning, 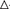
– reversal learning, 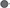
– sampling of brain tissue.

The scheme of behavioural experiments is presented on Figure 1.

#### Initial Learning for the Morris Water Maze

One hundred and sixteen rats were trained in the MWM spatial contextual learning model following standard procedures (Morris, 1984). The water maze was a circular tank (diameter, 160 cm; height, 60 cm) filled to a depth of 40 cm with milky water at 24°C. A hidden Plexiglas platform (10 cm × 10 cm) was submerged 2 cm below the water surface at the midpoint of one quadrant; the position of the platform remained the same throughout the training period. The maze was located in a test room containing several contextual visual marks.

Initially, rats were trained over five daily sessions (days 1–5), with four trials each day with a 60-s intertrial interval. Each trial lasted a maximum of 60 s and the escape latency to platform was recorded. At the start of all trials, the rats were placed in the pool at one of four starting positions. Rats that failed to find the platform within 60 s were softly guided there by the investigator. Animals remained on the platform for 30 s.

#### Repeated Learning in the Morris Water Maze

One cohort of trained rats (*n* = 65) was then subjected to repeated learning that started 14 days after the end of initial learning and lasted 4 days (days 19–22, four trials each day), with the same platform location and contextual stimuli as the initial learning.

#### Reversal Learning in the Morris Water Maze

Another cohort of trained rats (*n* = 28) was subjected to reversal learning on experimental days 19–22, with the location of the platform in the opposite quadrant relative to the initial learning, but the same contextual stimuli.

### Biochemical Analysis

#### Brain Tissue Preparation

The rats were decapitated with a guillotine 3 days after the second (*n* = 12), third (*n* = 8), or fifth (*n* = 5) training session. The control group included seven naïve rats. The choice of the time point for collecting brain tissue samples was carried out taking into account the need to minimise the effect of stress and motor activity on the cholinergic circuits and at the same time, based on the literature data on the dynamics of the systems consolidation and of the transition from resent memory to remote memory (Sutherland et al., 2010; Wartman and Holahan, 2013). The procedures regarding the preparation of sub-synaptic fractions from the brain structures and ChAT activity determination have been described previously (Zakharova et al., 2010a).

All preparative procedures were carried out at 2–4°C. The hippocampus and neocortex were separated from the brain and homogenised in an iso-osmotic solution. Both structures were isolated manually and taken as a whole. Approximate coordinates for hippocampus were AP-2 – AP-8, DV 0, ±6, H 2.5-0. The light and heavy synaptosomal fractions were isolated from each structure. The synaptosomal fractions were obtained from the rough mitochondrial fraction by centrifugation in a discontinuous sucrose gradient. The light synaptosomes were concentrated between the 1.0 and 1.2 M sucrose layers and the heavy synaptosomes were concentrated between the 1.2 and 1.4 M sucrose layers. The synaptosomes were precipitated and disrupted by combined shock procedures of suspension of the pellets in a hypo-osmotic solution and subsequent freeze-thaw exposure. The sub-fractions of synaptoplasm were obtained by centrifugation as supernatants from the disrupted synaptosomal fractions. The pellets of disrupted synaptosomes were resuspended in the hypo-osmotic solution and stratified on discontinuous sucrose gradients. After centrifugation of both the light and heavy disrupted synaptosomal fractions, the synaptic membrane sub-fractions were obtained in sucrose layers between 0.6 and 1.2 M. To achieve an iso-osmotic condition, the synaptic membrane sub-fractions were diluted with a solution containing 3 mM EDTA-Na_2_ and 9 mM Tris–HCl buffer, pH 7.4–7.5. All samples were stored at −80°C until the day of the assay.

#### Choline Acetyltransferase Assay

Choline acetyltransferase activity was determined by using Fonnum’s radiometric method (Fonnum, 1969), as described in detail in our earlier publication (Zakharova et al., 2010a). Briefly, the reaction contained 0.2 mM acetyl-CoA and [1-^14^C]-acetyl-CoA with SPA 5 mCi/mmol, 300 mM NaCl, 3 mM MgCl_2_, 0.2 mM physostigmine salicylate, 10 mM choline chloride, 0.5% Triton X-100, 0.5 mg/ml albumin from bull serum, 10 mM sodium phosphate buffer, 1 mM EDTA-Na_2_, pH 7.8 and 2.5–3.5 mg of the sub-fraction sample. The reaction was incubated in a water shaker at 37°C for 30–60 min. ACh synthesis was stopped by placing the mixture in an ice bath and adding an excess of ACh in the same sodium phosphate buffer. Then, sodium tetraphenylborate solution in butyl acetate was added and the mix was shaken vigorously in a shaker. The organic phase was gently separated from the inorganic phase by centrifugation. The organic phase with ACh was moved into scintillation liquid for organic solutions and the radioactivity of synthesised [^14^C]-ACh was quantified with a beta counter, determining the disintegrations per minute (DPM).

#### Reagents and Drugs

The following reagents were used: [1-^14^C]-acetyl CoA sodium salt from Amersham Pharmacia Bioscience; acetyl-CoA sodium salt, choline chloride, naphthalene, physostigmine salicylate, sucrose, tetraphenylborate sodium salt, Tris(hydroxymethyl)aminomethane sodium and salt from Sigma-Aldrich; ethylene glycol, sodium chloride, magnesium chloride monohydrate and sodium phosphate monobasic dihydrate from Merck; acetone, butyl acetate, dioxane, EDTA-Na_2_, PPO, POPOP, sucrose, toluol and some other reagents from REACHIM (Russian Federation).

### Statistical Analysis

#### Definition of Behavioural Variables

The escape latencies for every trial of the initial learning (days 1–5) and repeated or reversal learning (days 19–22) were analysed. These values were designated as idjs, where i is an ordinal number of the experimental day (d) and j is an ordinal number of trial (s) in the session. Moreover, the mean and standard deviation of escape latency in each session – idm and idSD, respectively, where i is an ordinal number of the experimental day (d) – was estimated for every animal and for the total cohort.

#### Analysis of Dynamics of Behaviour, Choline Acetyltransferase Activity and Their Associations

The analysis of variance (ANOVA) was applied to estimate the dynamics of the escape latency using experimental day as a Factor. When standard deviation was analysed, the daily average of latency was used as covariate.

The between-group differences in ChAT activity were estimated using the Wald–Wolfowitz criteria.

Spearman’s correlation analysis was performed to evaluate the associations between different performance measures as well as the associations between ChAT activity from different synaptosomal sub-fractions and the measures of initial learning. The individual scores of each Factor obtained using Principal Components Analysis (see below) also were included in correlation analysis. All statistical analyses were performed using STATISTICA 8 software (United States).

#### Principal Component Analysis

For additional analysis of the temporal structure of learning in the MWM, the 20 average escape latencies observed in 165 rats during the initial 5-day training were subjected to PCA with Varimax normalised rotation. The eigenvalue criterion (eigenvalue >1) was used to determine the appropriate number of factors. Items were allocated to factors according to their highest loading; loadings of 0.5 were considered as the threshold for interpretation of the factor. In addition, the individual scores of each factor were calculated.

## Results

### The Dynamics of Initial Learning

**FIGURE 2:**
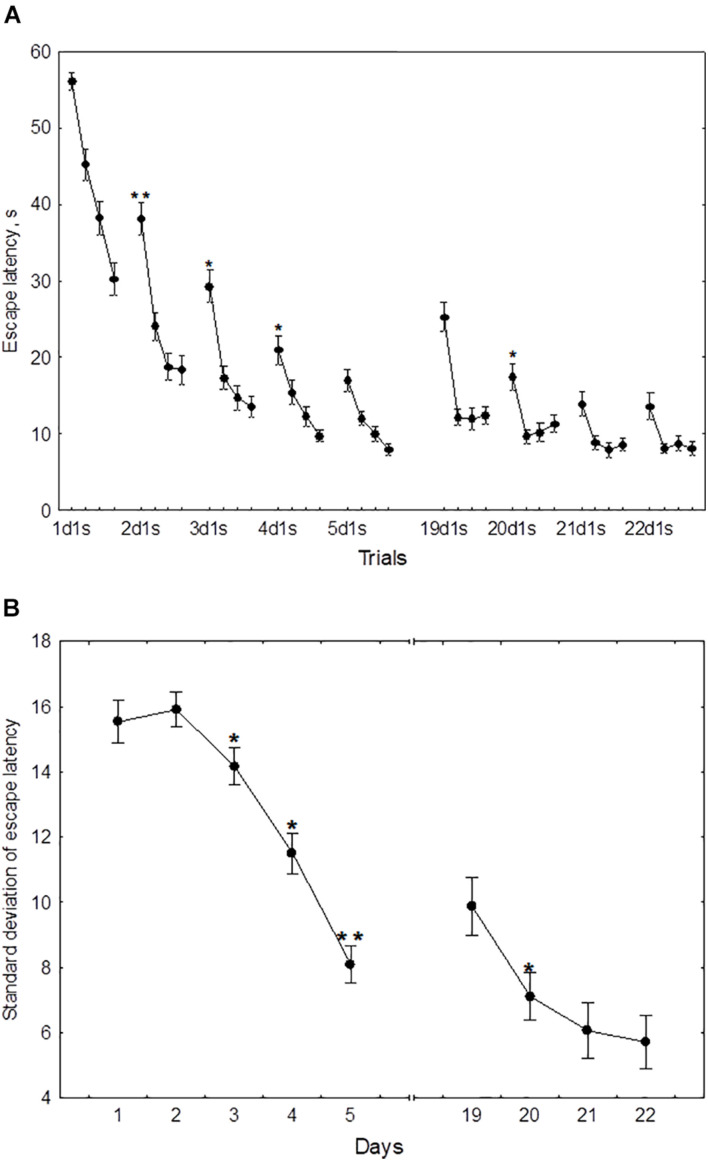
Mean **(A)** and standard deviation **(B)** of the escape latency for the initial learning (days 1–5) and the repeated learning (days 19–22). *p < 0.05 and **p < 0.01 relative to the previous day.

In the course of initial learning, both the daily mean escape latency and the escape latency in the first trial of each session declined from day 1 to day 4 and then reached a plateau (Figure 2A). Thus, the mean of 2dm was significantly lower than mean of 1dm [*F*(1,232) = 325.2, *p* < 0.001], the mean of 3dm was lower than the mean of 2dm [*F*(1,204) = 85.4, *p* < 0.001], and 4dm was lower than 3dm [*F*(1,188) = 4.45, *p* < 0.05]. The difference between 4dm and 5dm was not significant [*F*(1,178) = 2.01, *p* = 0.11].

Also, the mean of 2d1s was lower than 1d1s [*F*(1,232) = 119.3, *p* < 0.001], the mean of 3d1s was lower than 2d1s [*F*(1,204) = 34.1, *p* < 0.001], and 4d1s was lower than 3d1s [*F*(1,188) = 6.67, *p* < 0.05]. The difference between 5d1s and 4d1s was not significant [*F*(1,178) = 1.86, *p* = 0.13].

The standard deviation of the escape latency as a measure of performance instability in each session, declined from day 2 to day 5 (Figure 2B). Thus, the difference between 1dSD and 2dSD didn’t reach significance [*F*(1,232) = 0.91, *p* = 0.44], whereas 3dSD was significantly lower than 2dSD [*F*(1,204) = 3.92, *p* < 0.05], 4dSD was significantly lower than 3dSD [*F*(1,188) = 4.07, *p* < 0.05], and 5dSD was significantly lower than 4dSD [*F*(1,178) = 3.29, *p* < 0.05].

### Factor Structure of Initial Learning

A PCA using 20 mean escape latencies (days 1–5, four trials each day) revealed four factors explaining 50.6% of the total variance (Table 1). Factor 1 included the escape latency of trial 1d2s and trials 1–4 on day 2. Factors 2 and 4 were for the escape latencies on days 4–5 and day 3, respectively. Factor 3 included the escape latency of the first and third trials of day 1.

**TABLE 1:** Factor loadings of escape latencies during initial learning for the Morris water maze (MWM) in the four-factor model derived from the principal component analysis.

**Trials**	**Loading of escape latency**			
	**Factor 1**	**Factor 2**	**Factor 3**	**Factor 4**
1d1s	0.119907	0.040499	**−0.829473**	0.165782
1d2s	**0.540883**	0.001106	–0.035430	0.045726
1d3s	0.236944	0.034170	**0.593179**	0.354924
1d4s	0.466446	0.069060	0.250566	0.204827
2d1s	**0.682847**	–0.005547	–0.039499	0.065308
2d2s	**0.549670**	0.017067	0.048840	0.403527
2d3s	**0.635443**	0.380067	0.102547	–0.006406
2d4s	**0.558090**	0.270294	0.196961	0.214389
3d1s	0.478109	0.204500	–0.190799	0.487892
3d2s	0.071895	0.312702	0.054868	**0.616200**
3d3s	0.233446	0.106704	–0.057322	**0.663794**
3d4s	0.136853	0.146396	0.060678	**0.767604**
4d1s	0.117145	**0.502618**	–0.047546	0.394588
4d2s	0.028417	**0.787983**	–0.001680	0.194939
4d3s	0.085924	**0.781614**	–0.036480	0.038229
4d4s	0.344979	**0.537757**	0.091908	0.258714
5d1s	0.106231	**0.621901**	–0.047546	0.394588
5d2s	0.091281	**0.718345**	–0.001680	0.194939
5d3s	0.089291	**0.752671**	–0.036480	0.038229
5d4s	0.276147	**0.543281**	0.091908	0.258714

*The loadings values higher than 0.5 (the threshold level for Factor interpretation) are in bold.*

### Dynamics of Repeated Learning and Correlations With Initial Learning

During repeated learning, the escape latency in the very first trial (19d1s) and the mean latency of the first session were near the level that was reached on day 3 of initial learning (Figure 2A). Thus, the mean of 19d1s was lower than 1d1s [*F*(1,128) = 196.1, *p* < 0.001] and lower than 2d1s [*F*(1,128) = 25.3, *p* < 0.001], but didn’t differ from 3d1s [*F*(1,128) = 0.51, *p* = 0.62] and was higher than 4d1s [*F*(1,128) = 8.5, *p* < 0.01] and higher than 5d1s [*F*(1,128) = 16.9, *p* < 0.001]. Also, 19dm was lower than 1dm [*F*(1,128) = 326.5, *p* < 0.001] and lower than 2dm [*F*(1,128) = 101.3, *p* < 0.001], but didn’t differ from 3dm, 4dm, or 5dm [*F*(1,128) ≤ 3.1, *p* ≥ 0.21]. In the course of repeated learning, the mean of 20d1s declined relative to 19d1s [*F*(1,128) = 13.1, *p* < 0.01] and the mean of 20dm declined relative to 19dm [*F*(1,128) = 88.2, *p* < 0.001]. During 20–22 days these measures didn’t display significant changes [*F*(1,128) ≤ 2.9, *p* ≥ 0.29]. The mean of 19dSD was lower than 1dSD, 2dSD, and 3dSD [*F*(1,128) ≥ 32.9, *p* < 0.001] but didn’t differ from 4dSD to 5dSD [*F*(1,128) ≤ 1.1, *p* ≥ 0.29] (Figure 2B). The standard deviation of escape latency declined in the 20th day relative to 19th day [*F*(1,128) = 6.8, *p* < 0.05] and then remained stable [*F*(1,128) ≤ 2.9, *p* ≥ 0.23].

**FIGURE 3:**
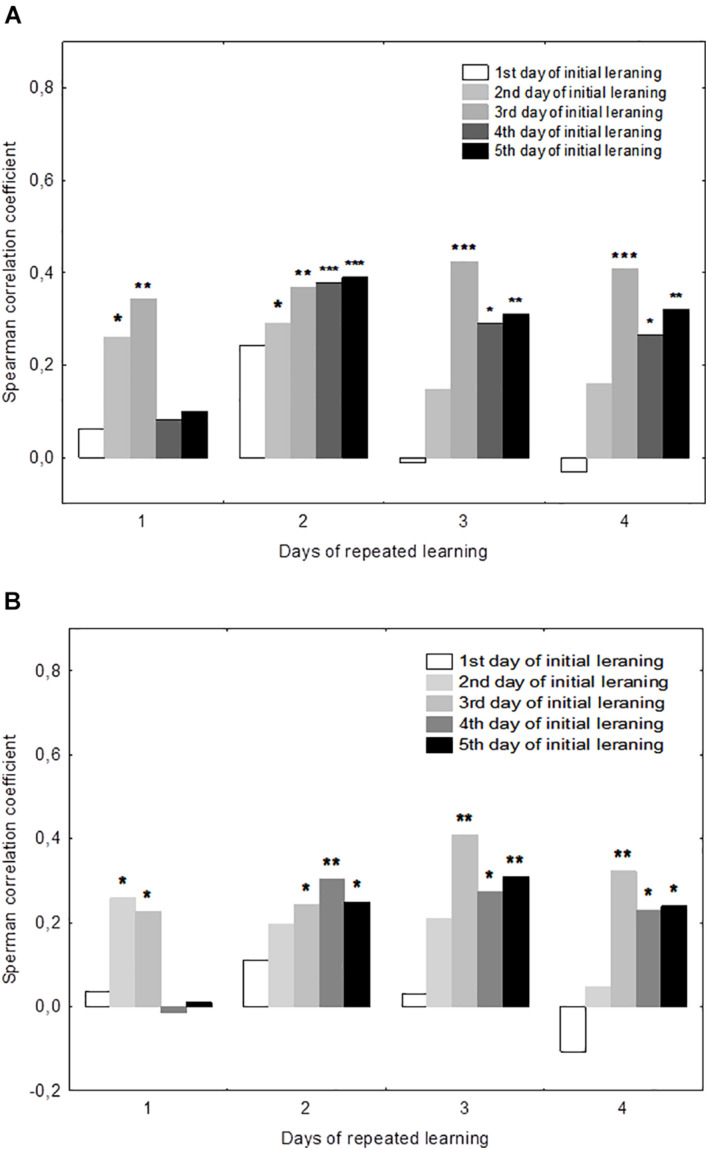
Correlations between performance measures in the initial and repeated learning. **(A)** Correlations between the daily mean escape latencies during repeated learning (days 19–22) and the daily mean escape latencies during initial learning (days 1–5). **(B)** Correlations between the escape latency for the first trial for each day of repeated learning (days 19–22) and the daily mean escape latencies during initial learning (days 1–5). *p < 0.05, **p < 0.01, and ***p < 0.001 indicate the significance of Spearmen’s correlations.

There were significant positive correlations of the performance on day 19 with performance on days 2–3 but not days 4–5 (Figure 3). This was true both for the mean escape latency on day 19 and for the 19d1s escape latency, which is a putative measure of remotely stored memory trace. The performance on days 20–22 but not on day 19 correlated positively with the performance on days 4–5. Thus, there was a correlation with performance on day 3 of initial learning for all days of repeated learning. The performance measures on day 2 displayed significant correlations with performance at the early stage of repeated learning and the performance measures on days 4–5 correlated positively with those observed at the later time points of repeated learning. Factor 4, including the performance measures on day 3 was the only factor that correlated positively with the escape latency of the first trial (*r* = 0.26, *p* = 0.023) and the mean latency of total session (*r* = 0.23, *p* = 0.041) on day 19.

### Dynamics of Reversal Learning and Correlations With Initial Learning

**FIGURE 4:**
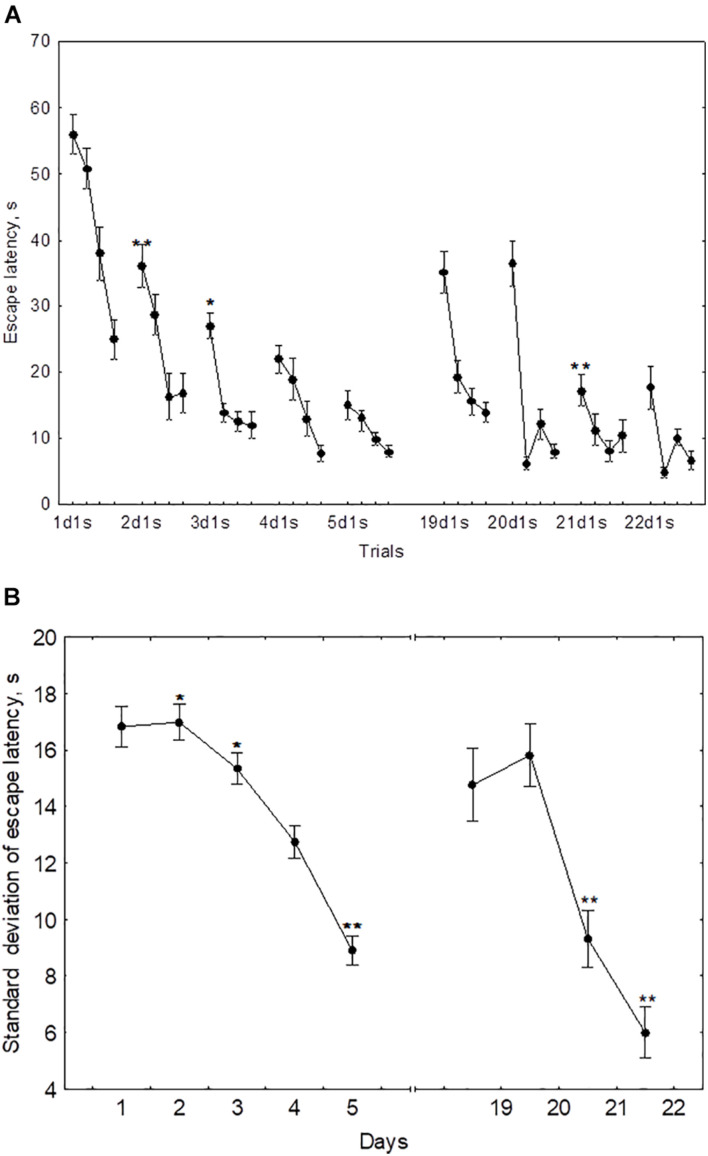
Mean **(A)** and standard deviation **(B)** of the escape latency to platform for the initial learning (days 1–5) and reversal learning (days 19–22). *p < 0.05 and **p < 0.01 relative to the previous day.

The performance dynamics of spatial navigation in cohort of rats subjected to reversal learning are presented in Figure 4. The escape latency of the first trial on the first day of reversal learning (19d1s), was lower than 1d1s [*F*(1,54) = 97.9, *p* < 0.001], didn’t differ from 2ds1 to 3ds1 [*F*(1,54) ≤ 1.39, *p* ≥ 0.18], and was higher than 4ds1 and 5ds1 [*F*(1,54) ≥ 7.12, *p* < 0.05] (Figure 4A). In the course of the first session of reversal learning, the escape latency declined about 3-fold from the first to the second trial [*F*(1,54) = 43.8, *p* < 0.001] and then remained relatively stable [*F*(2,54) ≤ 1.9, *p* ≥ 0.23]. However, the escape latency for the first trial of day 20 (20d1s) was again quite high and did not differ [*F*(1,54) = 0.8, *p* = 0.87] from the escape latency of the same trial on day 19 (19d1s). The escape latency for the first trial of day 21 (21ds1) was significantly decreased compared with the previous two sessions [*F*(1,54) ≥ 67.5, *p* ≤ 0.001].

The standard deviation of the escape latency for days 19–20 (reversal learning) didn’t differ from the level observed in days 1–2 [*F*(2,54) ≤ 1.1, *p* ≥ 0.492] and declined on days 21–22 [*F*(1,54) ≥ 42.5, *p* ≤ 0.001] (Figure 4B).

The mean escape latency of the second to fourth trials on day 19 (reversal learning) were correlated negatively with the mean escape latency on day 3 (*r* = −0.55, *p* = 0.041). The escape latency of the first trial and standard deviation of escape latency on day 20 displayed negative correlations with mean latency on day 3 (*r* = −0.73, *p* = 0.01 and *r* = −0.74, *p* = 0.01, respectively) and with Factor 4 (*r* = −0.60, *p* = 0.042 and *r* = −0.57, *p* = 0.026, respectively). No other correlations were significant.

### The Dynamics of Choline Acetyltransferase Activity

**FIGURE 5:**
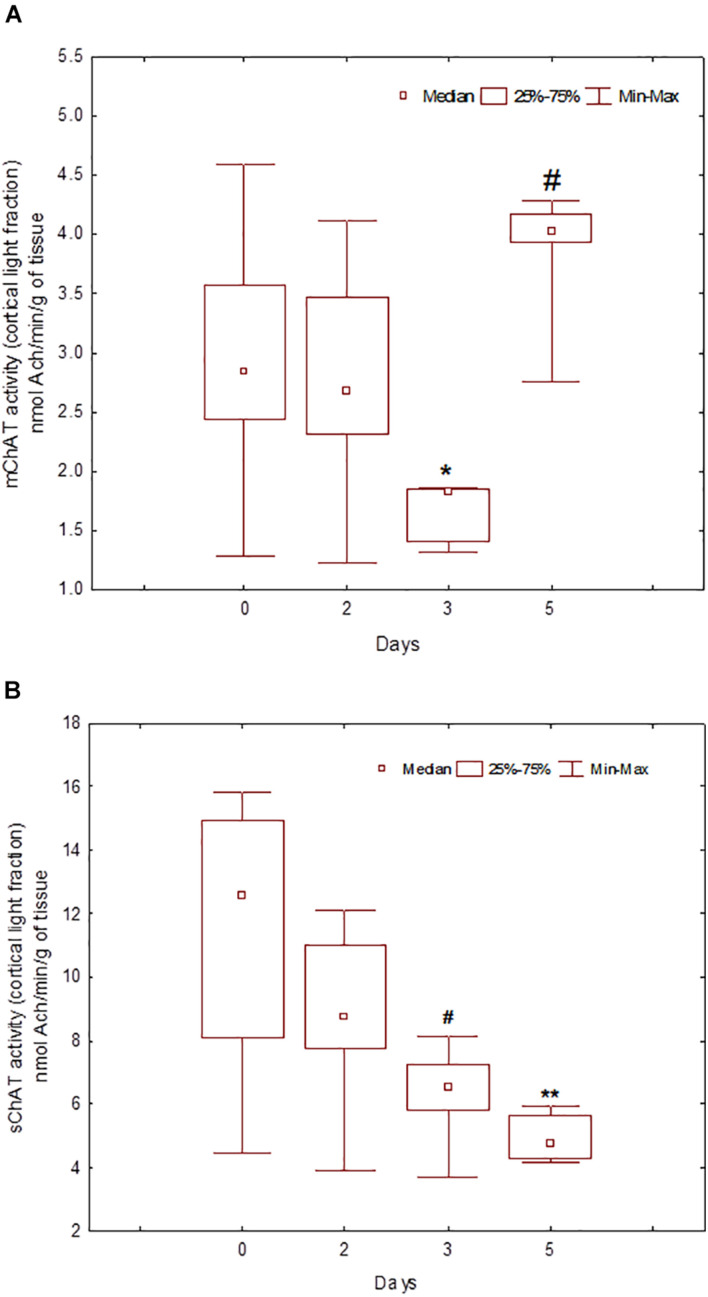
Choline acetyltransferase (ChAT) activity in light synaptosomal sub-fractions from the neocortex in naïve rats (day 0) and after 2, 3, or 5 days of training in the Morris water maze (MWM). The *y*-axis is the ChAT activity (nmoles ACh/min/g tissue). **(A)** mChAT from light synaptosomal fraction, **(B)** sChAT from light synaptosomal fraction, *p < 0.05 and **p < 0.01 relative to naïve rats. #*p* < 0.05 relative to the previous time point.

**FIGURE 6:**
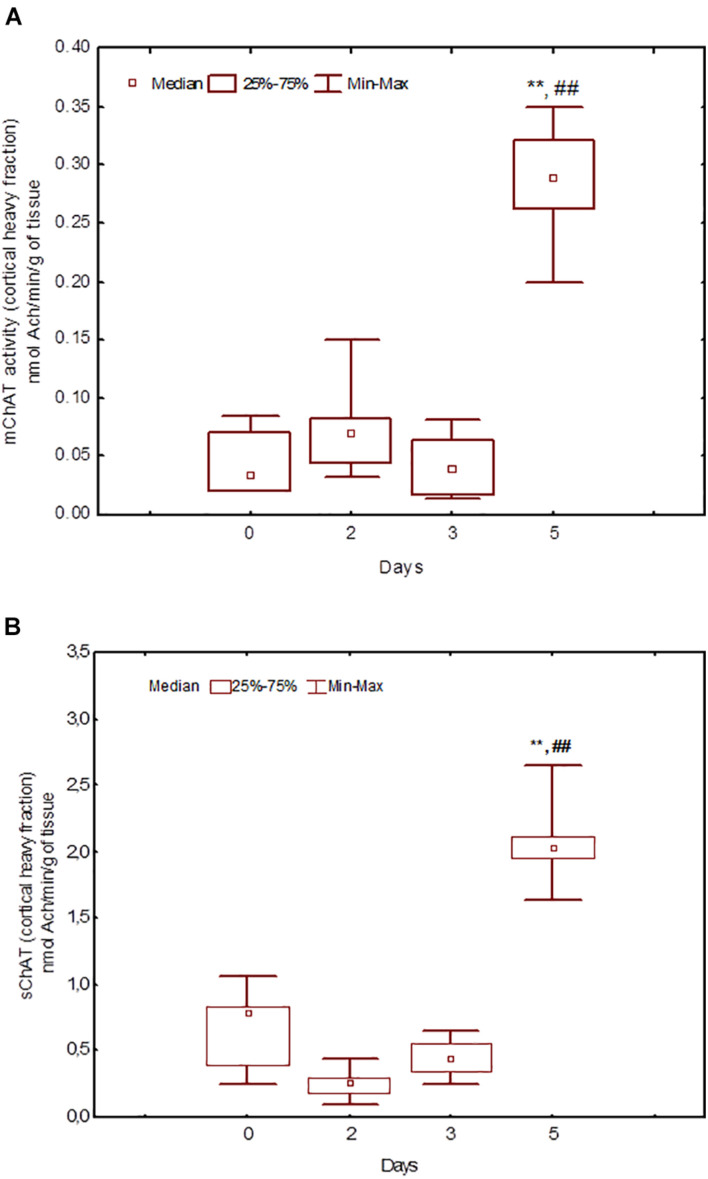
Choline acetyltransferase (ChAT) activity in heavy synaptosomal sub-fractions from the neocortex in naïve rats (day 0) and after 2, 3, or 5 days of training in the Morris water maze (MWM). The *y*-axis is the ChAT activity (nmoles ACh/min/g tissue). **(A)** mChAT from heavy synaptosomal fraction, **(B)** sChAT from heavy synaptosomal fraction, **p < 0.01 relative to naïve rats. ##*p* < 0.01 relative to the previous time point.

**FIGURE 7:**
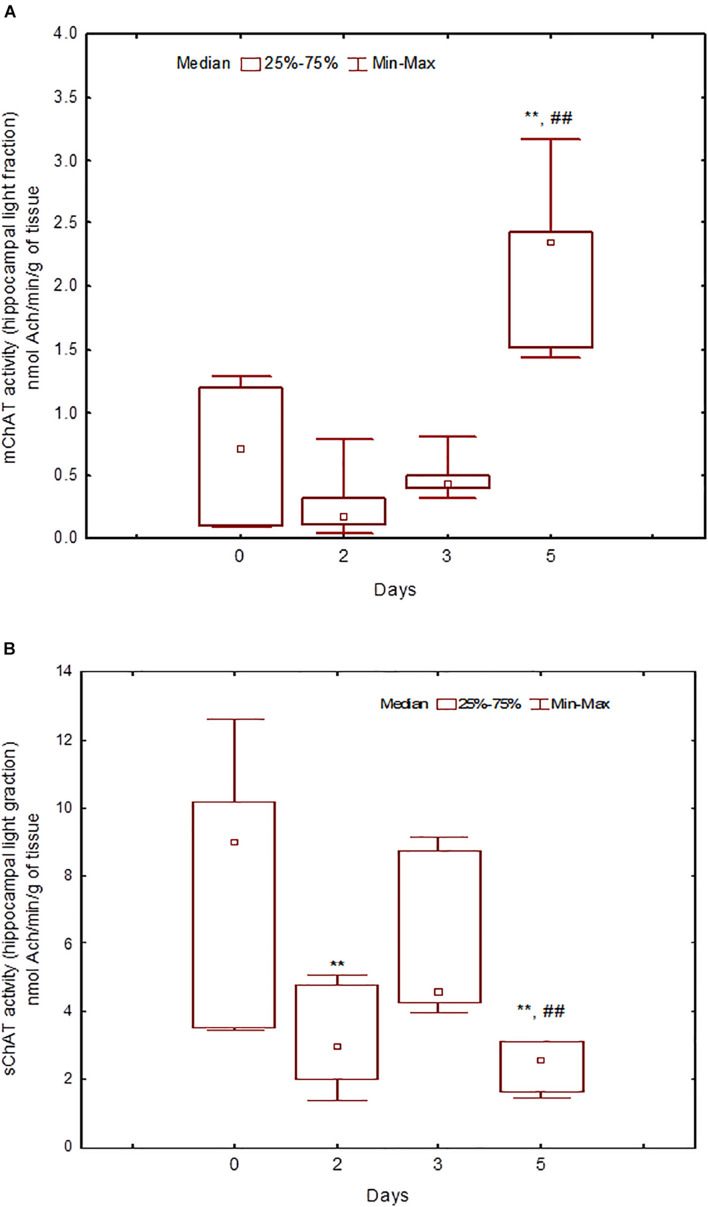
Choline acetyltransferase (ChAT) activity in light synaptosomal sub-fractions from the hippocampus in naïve rats (day 0) and after 2, 3, or 5 days of training in the Morris water maze (MWM). **(A)** mChAT from light synaptosomal fraction, **(B)** sChAT from light synaptosomal fraction, **p < 0.01 relative to naïve rats. ##*p* < 0.01 relative to the previous time point.

**FIGURE 8:**
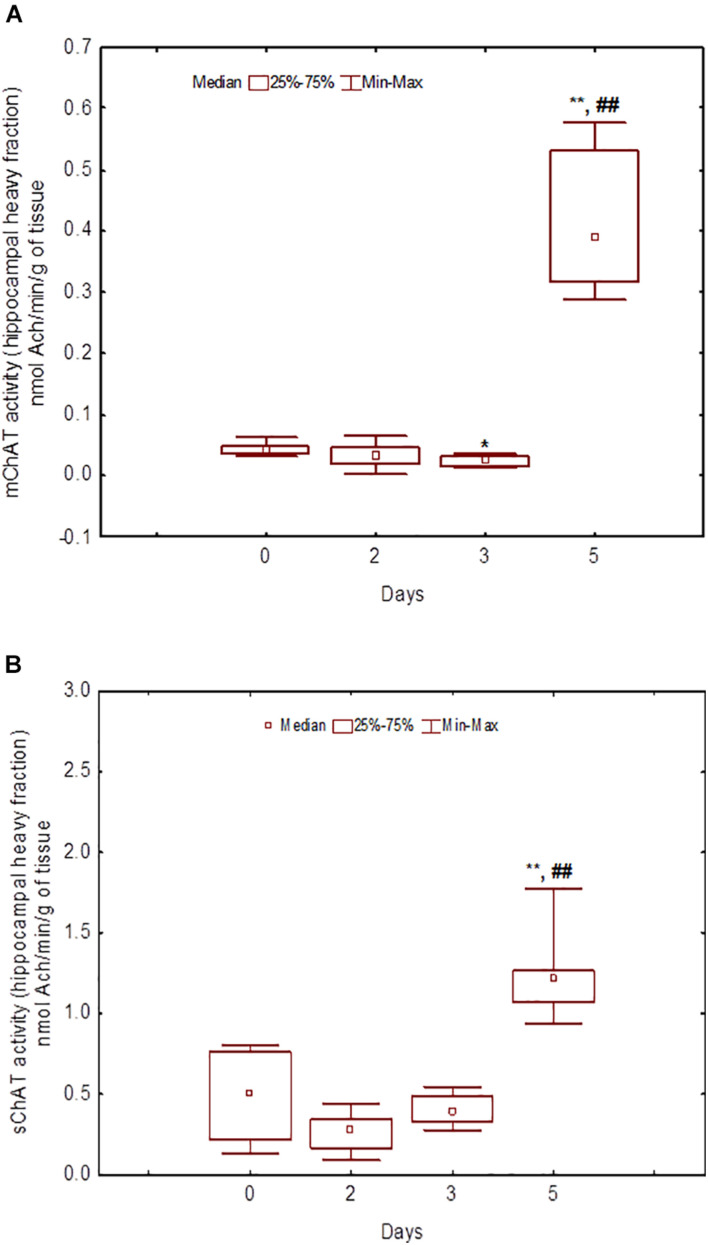
Choline acetyltransferase (ChAT) activity in heavy synaptosomal sub-fractions from the hippocampus in naïve rats (day 0) and after 2, 3, or 5 days of training in the Morris water maze (MWM). **(A)** mChAT from heavy synaptosomal fraction, **(B)** sChAT from heavy synaptosomal fraction, *p < 0.05 and **p < 0.01 relative to naïve rats. ##*p* < 0.01 relative to the previous time point.

During the training, the dynamics of ChAT activity depended on the compartmentalisation of the enzyme and synaptosome (Figures 5–8). In rats trained for 2 days, there was a decrease in sChAT activity from the hippocampal light fraction relative to the control level [*Z*(7,12) = 2.72, *p* = 0.006] (Figure 7B).

After 3 days of training, mChAT activity in the light cortical (Figure 5A) and heavy hippocampal (Figure 8A) synaptosomes decreased compared with the control levels [*Z*(7,8) = 2.72, *p* = 0.006 and *Z*(7,8) = 2.23, *p* = 0.025, respectively]. After 5 days of training, the sChAT activity in the light cortical (Figure 5B) and light hippocampal (Figure 7B) synaptosomes decreased compared with control levels [*Z*(7,5) = 2.35, *p* = 0.017 and *Z*(7,5) = 2.78, *p* = 0.004, respectively]. Of note, the activity of light cortical sChAT declined monotonically from day 2 to 5 (Figure 5B) whereas the activity of sChAT from the light hippocampal fraction displayed two-phase dynamics: (Figure 7B) it was lower compared with the control after 2 and after 5 but not after 3 [*Z*(7,8) = 1.21, *p* = 0.35] days of training. The mChAT activity from all fractions and the sChAT activity from cortical and hippocampal heavy synaptosomes increased significantly after 5 days of training [*F*(7,5) ≥ 2.84, *p* < 0.0025].

### Correlations Between Choline Acetyltransferase Activity and Behaviour

Correlation coefficients between performance measures and ChAT activity are presented in Table 2.

**TABLE 2:** Correlations between choline acetyltransferase (ChAT) activity and Morris water maze (MWM) performance in rats trained for 2, 3, or 5 days.

**Correlated measures**	**Valid *N***	**Spearman *R***	**t (N−2)**	***p*-level**
**Rats trained for 2 days**				
2d1s and ChAT cortical soluble light	12	–0.66	–2.75	0.02
2dm and ChAT cortical soluble light	12	–0.51	–1.90	0.08
2dSD and ChAT cortical soluble light	12	–0.85	–5.16	0.0004
**Rats trained for 3 days**				
2dm and ChAT cortical membrane light	8	–0.70	–2.21	0.078
2dSD and ChAT cortical membrane light	8	–0.78	–2.84	0.036
3dSD and ChAT cortical soluble heavy	8	–0.81	–3.38	0.015
3dm and ChAT hippocampal membrane heavy	8	–0.67	–2.19	0.071
3dSD and ChAT hippocampal membrane heavy	8	–0.76	–2.88	0.028
3d1s and ChAT hippocampal membrane heavy	8	–0.76	–2.88	0.028
2dm and ChAT hippocampal soluble heavy	8	0.77	2.74	0.041
2dSD and ChAT hippocampal soluble heavy	8	0.79	2.84	0.036
FACTOR 1 and ChAT hippocampal soluble heavy	8	0.89	4.43	0.007
1d2s and ChAT hippocampal soluble heavy	8	0.81	3.14	0.025
**Rats trained for 5 days**				
2dm and ChAT cortical membrane light	5	–0.90	–3.58	0.037
4dm and ChAT cortical soluble heavy	5	0.90	3.58	0.037
4dSD and ChAT cortical soluble heavy	5	0.90	3.58	0.037
5dSD and ChAT cortical soluble heavy	5	0.90	3.58	0.037
FACTOR 2 and ChAT cortical soluble heavy	5	0.90	3.58	0.037
FACTOR 4 and ChAT cortical soluble heavy	5	–0.90	–3.58	0.037

In rats trained for 2 days, the correlation analysis revealed a positive association between sChAT activity from the cortical light fraction and performance on day 2 (i.e., negative correlation with 2d1s and 2dSD escape latencies; the correlation with the 2dm was at the level of a trend). There were similar positive correlations between performance level and mChAT activity from the cortical light fraction on day 2 in rats trained for 3 or 5 days.

The ChAT activity from the hippocampal heavy fraction estimated after 3 days of training displayed the following pattern of associations with performance measures: high sChAT activity correlated with poor performance (including positive correlations with Factor 1 scores) on day 2 (previous day) and high mChAT activity correlated with successful performance on day 3 (current day).

For sChAT from the heavy cortical fraction, there was a positive association with performance stability on day 3 (i.e., a negative correlation with 3dSD). At the same time, after 5 days of training, higher sChAT activity was associated with worse spatial memory, especially with performance instability (i.e., 4dSD and 5dSz). Moreover, for light cortical sChAT activity, there was a positive correlation with Factor 2 scores and a negative correlation with Factor 4 scores.

## Discussion

The results of the present study evidenced the possibility to identify some relatively distinct stages of memory formation in the MWM and suggest the selective involvement of different cholinergic mechanisms in these stages. The results of factor analysis suggest at least three relatively distinct temporal stages of the elaboration of spatial navigation in the MWM in the context of the used protocol, namely days 1–2, day 3, and days 4–5. Our findings suggest a special significance of the performance on day 3 for the long-term stability of remotely stored memory trace. Indeed, the most stable positive correlations with the performance in the repeated learning sessions were for the measures of the performance on day 3 of initial learning and for Factor 4. Again, the performance in the initial stages of reversal learning displayed negative correlations only with the performance on day 3 of initial learning. Thus, successful partial space remapping negatively correlates with the stability of the stored navigation strategy as it was formed on day 3 of initial learning. As repeated learning continued, we observed a decline in correlations with performance on day 2 and an increase in correlations with performance on days 4–5 of initial learning. It is possible to speculate that on days 1 and 2 of initial learning, the automatic recording of attended sensory stimuli (Morris and Frey, 1997) is crucial while on day 3, the error-corrected way begins to dominate. The observed correlations evidenced that this error-corrected learning contributes most significantly to individual peculiarities of remotely stored memory trace. On days 4 and 5 of training, the integrated navigation system stabilised and the performance became partially automatic. This automation depends on executive control and is obviously partially innate. The fact that performance on days 4–5 of initial learning correlated with performance on days 20–21 but not day 19 of repeated learning supports this assumption.

Factor 1 loaded the escape latency for the second trial of day 1 (the putative measure of one-trial memory) and the escape latency for the day 2 trials. This finding suggests that some types of automatic sensory experience may be consolidated. Factor 3 included escape latencies for the first and third trials of day 1 but not subsequent days of initial learning. Hence, these trials are somewhat different than Factor 2 type of “quick” learning.

Our findings need to be verified in additional experiments including comparative analysis of repeated and reversal learning in large cohorts of rats after 2, 3, and 5 days of initial learning; estimation of the effects of various pharmacological treatments at these time points; and other manipulations. There are some data supporting the existence of the qualitatively different stages of spatial learning in the literature. Specifically, Berger-Sweeney et al. (1994) reported the selective involvement of cholinergic projections to the neocortex and hippocampus in different stages of spatial learning in the MWM using lesion techniques.

The data on the activity of different cholinergic systems at different time points of initial learning also suggest the presence of distinct stages of the memory trace formation. For the three studied time points, namely days 2, 3, and 5 of training in the MWM, sChAT and mChAT from cortical and hippocampal synaptosomes displayed distinct activity dynamics and specific correlations with behavioural measures.

sChAT from the light synaptosomal cortical fraction, which seems to include synaptosomes from basal forebrain projections, decreased from day 2 to 5 of training. For trial 1 of day 2, effective spatial navigation was associated with high activity of this enzyme. Together with our previous data (Zakharova et al., 2010a), these results suggest the contribution of baseline activity of cholinergic cortical projections into the inherited mechanisms of rapid spatial memory formation during the early learning stage. mChAT activity from the light cortical fraction declined after 3 days of training and increased again up to the control level after 5 days of training. On days 3 and 5, there was a positive association between high mChAT activity and successful performance on day 2. Thus, compared with sChAT, the correlation of the activity of mChAT from light cortical synaptosomes with behavioural measures appeared later (on day 3) and was more stable (i.e., was seen also in rats trained for 5 days).

Specific functions of sChAT and mChAT have been discussed in the literature (Cooper, 1994; Pyle et al., 2000; Pahud et al., 2003; Zakharova and Dudchenko, 2014). To bind to membrane structures, sChAT needs some modifications, in particular, phosphorylation of and binding with proteins in the plasma membrane (Pahud et al., 2003). According to some hypotheses, mChAT plays a role in ion-dependent mechanisms of rapid ACh synthesis and re-uptake that are connected with increased efficacy of cholinergic synaptic transmission (Cooper, 1994; Pyle et al., 2000; Zakharova and Dudchenko, 2014). Thus, together with the data from the literature, our results suggest that initial activity of cholinergic projections to the neocortex is important for the early stage of spatial learning. On the other hand, mechanisms participating in the regulation of the mChAT activity from cortical projections (phosphorylation, etc.) are involved in spatial memory consolidation at the transition from early automatic sensory processing to the dominance of the error-corrected learning paradigm.

After 3 days of training – the stage that according to our results, is key for shaping the remotely stored memory trace – most correlations were found for ChAT activity from heavy hippocampal synaptosomes. In rats trained for 3 days, the sChAT activity displayed negative correlations with spatial navigation on the previous day (day 2) whereas mChAT activity correlated positively with performance on the current day (day 3). These results suggest that the hippocampal cholinergic neurons comprising the heavy synaptosomes could participate in the integration of ongoing day 3 (mainly error-corrected) learning and information obtained in the earlier stage (day 2), which engages mainly the automatic sensory processing. Some data from the literature allow us to propose that this fraction contains the pre-synapses from intrinsic hippocampal neurons (Mukhin et al., 2002). Despite the small population of cholinergic interneurons in the hippocampus, they exhibit notable heterogeneity, including different mechanisms of interaction with neurons that utilise other neurotransmitters (Frotscher et al., 2000; Yi et al., 2015). In any case, according to our data, hippocampal cholinergic neurons that are the source of heavy synaptosomes play a prominent role in the formation of spatial memory at the temporal stage that is the most important for the shaping of remotely stored memory trace.

As in the case of projection cortical neurons, in interneurons of the hippocampus, the activity of soluble ChAT displayed correlations with the performance measures in the early stage of training, while the activity of the membrane-bound form of the enzyme correlated with the performance on the later stage. However, in contrast to ChAT from cortical projections, the correlation of the enzyme activity from hippocampal interneurons with the efficiency of performance in MWM, was observed only in animals trained for 3 days. In addition, the correlations with performance were positive for membrane-bound form and negative for soluble one.

Based on the pattern of correlations between ChAT activity in hippocampal interneurons and behaviour, it can be assumed that the processes that affect the activity of sChAT are involved in the early stages of spatial memory consolidation. In particular, a prolonged search for a platform in animals with poor performance on the second day could stimulate the expression of sChAT. At the same time, successful performance on the third day may be associated with the mechanisms that increase the activity of mChAT. These assumptions can be verified in the further studies using sample clustering and subsequent assessment of correlation patterns in “good” and “poor” learners.

Taken together, our results revealed that the most prominent role of cortical cholinergic projections occurred at the early stage of spatial learning while later the hippocampal intrinsic cholinergic neurons became the key players. This is in agreement with previous data about the effects of lesions of hippocampal and cortical cholinergic circuits. Thus, in the experiments with lesion of the nucleus basalis magnocellularis, the main source of cholinergic projections to the cortex, substantial impairment of performance in the MWM began on the first training session (Nieto-Escámez et al., 2002). At the same time, in rats with lesions of the medial septum, worsened performance became apparent after day 3 (Kang et al., 2006). These data, alongside our present results, could be regarded as evidence of the selective involvement of cholinergic hippocampal intrinsic neurons in some stages of the formation of spatial memory trace.

The pattern of associations between ChAT activity and behaviour observed in rats trained for 3 days is in agreement with our previous data (Zakharova et al., 2010a). It should be noted that in the above-mentioned study, the training took place in a pool with a smaller diameter (120 cm), so the asymptotic level of the escape latency had been reached earlier and the onset of various stages could have been shifted in time relative to the those reported in the present study. Nevertheless, similarly to the present study, successful performance at the beginning of training displayed an association first with high sChAT and mChAT activities from the cortical light fraction and later with ChAT from the heavy hippocampal fraction.

For sChAT activity from the hippocampal light fraction (likely containing synaptosomes from projection fibres), no correlations with spatial performance in any training session has been revealed. This finding seems to be in agreement with the concept assuming that the activity of the cholinergic projections to hippocampus is not critical for spatial learning (Berger-Sweeney et al., 1994; Parent and Baxter, 2004). It should be noted that a significant amount of literature data contradicts this hypothesis. However, these studies report both positive and negative associations between the activity of the septohippocampal cholinergic system and the measures of learning and memory (Ang et al., 2006; Gökçek-Saraç et al., 2013; Kolisnyk et al., 2013). In this regard, attention should be paid to the two-phase dynamics of the activity of soluble ChAT of septohippocampal projections observed in our study. In particular, its significant decrease is observed after the 2nd and 5th days of training. These time points can coincide with different stages of spatial memory consolidation, the mechanisms for which, as shown earlier, require a decrease in the activity of the ACh system (Hasselmo and McGaughy, 2004). Thus, the absence of correlations in this case may be due to the non-linearity of associations between the activity of septohippocampal cholinergic projections and memory mechanisms.

After 5 days of learning, there was only an association between behavioural measures and sChAT activity from heavy synaptosomal cortical fraction, which seems to include synaptosomes from cortical interneurons (Mukhin et al., 2002). The existence of cholinergic cortical interneurons is well established, and many populations of these cells were shown to excrete GABA and other transmitters or modulators together with ACh (Dudai et al., 2020). Of note, in rats trained for 3 days, the high sChAT activity was associated with stable performance (low standard deviation), but after 5 days of training the correlation was the opposite: high enzyme activity was associated with unstable performance (high standard deviation of the escape latency) on days 4 and 5. The correlations with Factors 2 and 4 (including the escape latencies from day 4 and day 3, respectively) also displayed opposite signs. It can be assumed that in rats with successfully formed error-corrected remotely stored learning rule (which is elaborated mainly on day 3 of training), the increased sChAT activity from cortical heavy synaptosomes observed on day 5 is important for mechanisms of executive control of automated spatial habit and supports its adaptive flexibility.

The involvement of cortical cholinergic circuits into mechanisms of memory trace flexibility has been shown in many studies (for a review, see Prado et al., 2017). Our data allow to propose the important role of cholinergic cortical intrinsic neurons in executive control and behaviour flexibility.

On the other hand, our data are in some degree consistent with the results obtained by Obermayer et al. (2019) indicating that the activity of cholinergic cortical interneurons is important for the successful performance of the spatial memory task in the radial maze in the late stage of training but not in initial one. It can be assumed that the activation of these neurons provides the optimal level of attention for successful retrieval of spatial memory, in particular in animals with poor memory trace. To verify any of these assumptions, further studies are needed with the inclusion of a large number of animals with significant differences in the level of performance of the spatial task at the late stages of training.

Previously, studies on ChAT activity in homogenised brain tissue from naive animals revealed its association with spatial memory in MWM only in old rats (Dunbar et al., 1993). At the same time, total ChAT activity did not differ in young adult and aged animals. Our results showed that more precise estimation of the different types of enzymes as well as selective study of different stages of memory formation could help to identify more associations.

Together with the relatively modest number of rats in some of the groups used to evaluate ChAT activity, one of the limitations of the present work is the use of a single statistical approach for the selection of time points for ChAT analysis. For more robust conclusions, the application of functional ablation or stimulation techniques is necessary. Nevertheless, the approach used can be regarded as one of the possible ways (or the first step in the direction) of delineating temporal stages of spatial learning and could be applied in the studies of other signalling pathways.

## Data Availability Statement

The raw data supporting the conclusions of this article will be made available by the authors, without undue reservation.

## Ethics Statement

The animal study was reviewed and approved by the Ethical Committee of P.K. Anokhin Institute of Normal Physiology.

## Author Contributions

All authors listed have made a substantial, direct and intellectual contribution to the work, and approved it for publication.

## Conflict of Interest

The authors declare that the research was conducted in the absence of any commercial or financial relationships that could be construed as a potential conflict of interest.

## Publisher’s Note

All claims expressed in this article are solely those of the authors and do not necessarily represent those of their affiliated organizations, or those of the publisher, the editors and the reviewers. Any product that may be evaluated in this article, or claim that may be made by its manufacturer, is not guaranteed or endorsed by the publisher.
